# Alzheimer resemblance atrophy index, BrainAGE, and normal pressure hydrocephalus score in the prediction of subtle cognitive decline: added value compared to existing MR imaging markers

**DOI:** 10.1007/s00330-022-08798-0

**Published:** 2022-04-29

**Authors:** Panteleimon Giannakopoulos, Marie-Louise Montandon, François R. Herrmann, Dennis Hedderich, Christian Gaser, Elias Kellner, Cristelle Rodriguez, Sven Haller

**Affiliations:** 1 Department of Psychiatry, University of Geneva, Geneva, Switzerland; 2 Medical Direction, University of Geneva Hospitals, Geneva, Switzerland; 3 Department of Rehabilitation and Geriatrics, Geneva University Hospitals and University of Geneva, Geneva, Switzerland; 4 Department of Diagnostic and Interventional Neuroradiology, Klinikum rechts der Isar, School of Medicine, Technical University of Munich, Munich, Germany; 5 Departments of Psychiatry and Neurology, Jena University Hospital, Jena, Germany; 6 Medical Physics, Department of Radiology, Medical Physics, Medical Center – University of Freiburg, Faculty of Medicine, University of Freiburg, Freiburg, Germany; 7 CIMC - Centre d’Imagerie Médicale de Cornavin, Geneva, Switzerland; 8 Department of Surgical Sciences, Radiology, Uppsala University, Uppsala, Sweden; 9 Faculty of Medicine of the University of Geneva, Geneva, Switzerland; 10 Department of Radiology, Beijing Tiantan Hospital, Capital Medical University, Beijing, China

**Keywords:** Alzheimer, BrainAGE, Normal-pressure hydrocephalus, MTA, Microbleeds

## Abstract

**Objectives:**

Established visual brain MRI markers for dementia include hippocampal atrophy (mesio-temporal atrophy MTA), white matter lesions (Fazekas score), and number of cerebral microbleeds (CMBs). We assessed whether novel quantitative, artificial intelligence (AI)–based volumetric scores provide additional value in predicting subsequent cognitive decline in elderly controls.

**Methods:**

A prospective study including 80 individuals (46 females, mean age 73.4 ± 3.5 years). 3T MR imaging was performed at baseline. Extensive neuropsychological assessment was performed at baseline and at 4.5-year follow-up. AI-based volumetric scores were derived from 3DT1: Alzheimer Disease Resemblance Atrophy Index (AD-RAI), Brain Age Gap Estimate (BrainAGE), and normal pressure hydrocephalus (NPH) index. Analyses included regression models between cognitive scores and imaging markers.

**Results:**

AD-RAI score at baseline was associated with Corsi (visuospatial memory) decline (10.6% of cognitive variability in multiple regression models). After inclusion of MTA, CMB, and Fazekas scores simultaneously, the AD-RAI score remained as the sole valid predictor of the cognitive outcome explaining 16.7% of its variability. Its percentage reached 21.4% when amyloid positivity was considered an additional explanatory factor. BrainAGE score was associated with Trail Making B (executive functions) decrease (8.5% of cognitive variability). Among the conventional MRI markers, only the Fazekas score at baseline was positively related to the cognitive outcome (8.7% of cognitive variability). The addition of the BrainAGE score as an independent variable significantly increased the percentage of cognitive variability explained by the regression model (from 8.7 to 14%). The addition of amyloid positivity led to a further increase in this percentage reaching 21.8%.

**Conclusions:**

The AI-based AD-RAI index and BrainAGE scores have limited but significant added value in predicting the subsequent cognitive decline in elderly controls when compared to the established visual MRI markers of brain aging, notably MTA, Fazekas score, and number of CMBs.

**Key Points:**

• *AD-RAI score at baseline was associated with Corsi score (visuospatial memory) decline.*

• *BrainAGE score was associated with Trail Making B (executive functions) decrease.*

• *AD-RAI index and BrainAGE scores have limited but significant added value in predicting the subsequent cognitive decline in elderly controls when compared to the established visual MRI markers of brain aging, notably MTA, Fazekas score, and number of CMBs.*

**Supplementary Information:**

The online version contains supplementary material available at 10.1007/s00330-022-08798-0.

## Introduction

Using magnetic resonance (MR) neuroimaging to predict subsequent cognitive decline is a main issue in Alzheimer’s disease (AD) research. Several neuroimaging markers have been proposed in clinical routine, including hippocampal atrophy assessed using the semiquantitative visual reading mesio-temporal atrophy (MTA) score [1], T2w hyperintense white matter lesions (WML) using the Fazekas score [2], and number of cortical microbleeds (CMB) in community- and hospital-based cohorts. MTL atrophy allowed for identifying patients at high risk for Alzheimer type dementia among those with minor cognitive impairment (for review, see [3]). In the same line, longitudinal studies suggested a negative effect of WMH presence and progression on general intelligence, attention, and executive functions in non-demented elders [4–6]. Three population-based studies (Rotterdam [7], Framingham Heart [8], and AGES-Reyjkavik [9]) also supported the deleterious effect of CMB on cognition in non-demented elders.

While the diagnostic performance of these classic MR markers is well-established in patients with already present cognitive decline, their value in predicting subtle cognitive decline in healthy elderly individuals remains a matter of debate [10]. MTA, cortical microbleeds, and WML have all been thought to impact cognitive performance at the pre-mild cognitive impairment (MCI) state [11–16]. However, negative or ambiguous data were also frequently reported [17–19]. This raises the question of whether more recently proposed artificial intelligence (AI) MR imaging markers might have an added value in predicting subsequent cognitive decline in healthy elderly controls. The Alzheimer Disease Resemblance Atrophy Index (AD-RAI) is an AI-based metric derived from 3DT1 volumetric brain MRI [20–23]. In the meantime, the concept has also been adopted by commercial vendors. This operator-independent AD-RAI encapsulates hippocampal atrophy as well as atrophy of the established AD signature regions [24]. In a totally different perspective, the Brain Age Gap Estimate (BrainAGE) score is based on a machine learning regression task and has been proposed to capture global deviations from normal brain aging [25–27]. In a first step, associations between brain MRI and patient age are learned by an algorithm and then applied to new individuals in order to predict their age based on his or her brain MRI (usually 3D T1-weighted scans serve as input). Contrasting predicted and chronological brain age can either result in expected (predicted age ~ chronological age), advanced (predicted age > chronological age), or delayed (predicted age < chronological age) brain aging [25]. Both parameters are defined based on structural brain MRI atrophy patterns. The normal pressure hydrocephalus NPH index is another AI calculated marker derived from volumetric 3D T1 images, which indicates the degree of an NPH configuration in elderly cohorts [28]. Definite full (NPH) fulfilling diagnostic and clinical criteria remains rare in cognitively preserved elderly individuals. In the context of healthy elderly individuals, we consider NPH configuration as a spectrum, rather than a binary variable. Some participants may display beginning imaging features of NPH configuration, without fulfilling the clinical diagnosis of NPH. Considering currently emerging theories, impairment of CSF flow (resulting in diminished clearance) may be another factor contributing to cognitive decline, which might confound clinical and biomarker interpretation in AD [29, 30].

The main purpose of the present study is to assess the added value of the AI-based parameters AD-RAI, BrainAGE, and NPH index, as compared to the established visual imaging markers (MTA, Fazekas, and number of CMB), in the prediction of early cognitive decline in healthy elderly individuals. We had the opportunity to investigate these imaging parameters at baseline in a community-dwelling sample of 80 cognitively intact elderly individuals who were followed up longitudinally during a 4.5-year period.

## Materials and methods

### Participants

The study was approved by the local ethics committee and all participants gave written informed consent prior to inclusion. The selection of cases among participants of a still ongoing cohort study aiming to identify predictive biomarkers of subtle cognitive decline among healthy elders was described in detail elsewhere [31]. Briefly, the present cohort included only healthy controls with preserved cognition; no history of psychiatric, neurological, and major medical conditions; and no regular use of psychotropic medication [16, 31–33]. All cases were recruited via advertisements in local newspapers and media. The cohort included elderly Caucasian white individuals living in Geneva and Lausanne catchment area. Due to the need for excellent French knowledge (to participate in detailed neuropsychological testing), most of the participants were Swiss (or born in French-speaking European countries, 92%). Substantial vascular burden as evidenced by subtle cardiovascular symptoms, hypertension (non-treated), and a history of stroke or transient ischemic episodes was an additional exclusion criterion. All cases included in this study had three neuropsychological evaluations (baseline, 18 months, and 54 months), structural brain MRI at baseline, APOE genotyping, and amyloid and FDG PET at last follow-up. Our sample included 80 individuals (46 females, age range 73.4 ± 3.5 years).

### Neuropsychological assessment

At baseline, all individuals were evaluated with a neuropsychological battery including the MMSE score as well as one neurocognitive test for the main cognitive functions as follows: Trail-Making test A (attention), Trail-Making test B (executive functions), Digit Span (working memory), Corsi (visuospatial memory), and Shapes test (episodic memory) described in details previously [16, 31–34]. For each neuropsychological testing, the final score of change between inclusion and last follow-up was defined as the sum of the changes observed at the two follow-ups as previously described. The 0 cut-off was subsequently used to identify decliners (< 0) from non-decliners (≥ 0) for the binary classification used in the subsequent statistical analysis.

### MR imaging

At baseline, imaging data were acquired on a 3-T MRI scanner (TRIO SIEMENS Medical Systems). The structural high-resolution T1-weighted anatomical scan was performed with the following fundamental parameters: 256 × 256 matrix, 176 slices, 1 mm isotropic, TR = 2300 ms, TE 2.27 ms; axial T2w sequences: 512 × 310 matrix, 30 slices, 4 mm thickness, TR 4000 ms, TE 105 ms; susceptibility-weighted imaging (SWI): 256 × 208 matrix, 128 slices, TR 28 ms, TE 20 ms); pulsed ASL: 64 × 64 matrix, 20 slices, 6 mm thickness, TR 4000 ms, TE 12 ms, inversion time 1800 ms.

### Visual MR analysis

The visual analysis of brain MR images was described in detail previously [16, 31] and was assessed by an experienced neuroradiologist (20 years of experience). Briefly, MTA was assessed at baseline according to the established score [35], ranging from 0 (no atrophy) to 4 (significant atrophy). The MTA score is a simple and clinically established semi-quantitative scale and was analyzed on the 3DT1 scan.

WMH load at baseline was assessed according to the established Fazekas score [2] ranging from 0 (no white matter lesions) to 3 (confluent white matter lesions) based on the axial T2w scan. Like the MTA score described above, the Fazekas score is a simple and widely used tool to assess the severity of white matter damage in brain aging.

The number of CMB was assessed based on the SWI sequences. Only lesions were considered which are probable CMB, and the corresponding phase images were also analyzed to discriminate probable CMB versus micro-calcifications [36, 37]. At baseline, the total number of CMB and number of CMB per location (supratentorial superficial, supratentorial deep, and infratentorial) were evaluated.

### Image pre-processing

The structural T1-images were segmented into gray matter, white matter, and cerebrospinal fluid (CSF) using SPM12 (Welcome Trust Center for Neuroimaging, warped into the MNI (Montreal Neurological Institute) space (using modulation of gray value by the Jacobian of the warp) and smoothed by full width-half-max 3mm filter, like what is done for usual voxel-based morphometry analyses. The segmentation results have been inspected visually prior to further processing, none of the cases had to be excluded because of obvious failures.

### Automatic assessment of AD-RAI

Automated assessment of the AD-RAI was performed using a voxel-based support-vector-machine-learning approach (SVM). The basic principle is described in [20]. The smoothed segmentations of gray matter and CSF are used as direct inputs to an SVM. In the context of this work, a customized research version of the “VEOmorph” Software (www.veobrain.com) was trained with data from another study (in total 741 subjects, 445 Healthy Controls, 234 AD, 39 FTLD, 23 LB). The age distribution in the training (range 45–90 years, median value 73 +/− 8.2 years) covers the range of the population in the present study Ground truth labelling was based on clinical diagnoses. Fivefold cross-validation was performed. The AD-RAI was determined as the probability score of the SVM output as a value between 0 and 1. The trained algorithm was applied to the current dataset without any further adjustments.

### Automatic assessment of BrainAGE

We used a modified approach to our pre-processing as described previously [25]. T1-weighted images were pre-processed using the CAT12 toolbox (http://www.neuro.uni-jena.de/cat) and the SPM12 software (http://www.fil.ion.ucl.ac.uk/spm/software/spm12), running under MATLAB (www.mathworks.com). To train the age estimation framework, we used MRI data of 547 healthy subjects (242 male) from the publicly accessible IXI cohort (http://brain-development.org/ixi-dataset/), aged 19–86 years (mean (SD) = 48.1 (16.6) years). In brief, the BrainAGE framework utilizes relevance vector regression (RVR) using a linear kernel and a linear combination of pre-processed GM and WM images [38, 39]. The difference between estimated and chronological age yields the individual brain age gap estimation (BrainAGE) score, with positive values indicating accelerated and negative values indicating decelerated structural brain aging. Recent work has demonstrated that this method provides reliable and stable estimates of BrainAGE at a mean absolute error of 3.322 years, rendering this framework at least equal to several recently introduced deep learning algorithms [40].

### Automatic assessment of NPH score

The NPH score was calculated as described in [28]. A support vector machine (SVM) was trained in 30 NPH patients treated with ventriculoperitoneal shunts and 30 healthy controls, with the smoothed segmentations of gray matter and CSF as inputs. The output is a NPH probability score between 0 and 100%.

### Amyloid PET imaging

18F-Florbetapir (Amyvid- and 18F-Flutemetamol-PET (Vizamyl) data were acquired on 2 different tomographs (Siemens Biograph^TM^ mCT and GE Healthcare Discovery PET/CT 710 scanners) of varying resolution and following different platform-specific acquisition protocols. The 18F-Florbetapir images were acquired 50 to 70 min after injection and the 18F-Flutemetamol images 90 to 120 min after injection. PET images were reconstructed using the parameters recommended by the ADNI protocol aimed at increasing data uniformity across the multicentre acquisitions. More information on the different imaging protocols for PET acquisition can be found on the ADNI website (http://adni.loni.usc.edu/methods/).

### Visual amyloid PET analysis

The visual analysis of amyloid PET images was conducted by an independent, board-certified specialist in nuclear medicine (V.G.) blind to the neuropsychological data, following the tracer-specific standardized operating procedures approved by the European Medicinal Agency. Specifically, regional positivity was assessed for each scan, specifying if uptake was identified in the lateral frontal, parietal, posterior cingulate and precuneus, anterior cingulate, temporal lateral, and striatal regions in either of the two hemispheres [41].

### FDG PET imaging

PET/CT data acquisition was performed on a Siemens Biograph^TM^ mCT or Vision scanner according to the guidelines of the European Association of Nuclear Medicine (EANM) [64]. The PET acquisition was started approximately 30 min after injection of 200 MBq of 18F-FDG. The PET emission study (20 min, one-bed position) was conducted followed immediately by the CT study used for attenuation correction. Ultra-law dose brain CT imaging was performed under standard conditions (120 kVp, 20 mAs, 128 Å ~ 0.6 collimation, a pitch of 1 and 1 s per rotation).

### Visual FDG PET analysis

FDG PET reading was performed by visual analysis of the output of an automated voxel-wise comparison with a reference database, as recommended in guidelines [42] and previously described in detail [43]. Images were classified as normal when no significant deviations from the normal distribution were observed and pathological when significant regional reductions in glucose metabolism were documented.

### Statistical analysis

Gender-related differences in demographic, neuropsychological, and imaging data were assessed with chi^2^, Mann-Whitney u test, and unpaired t-test depending on the variable distribution. Multiple logistic regression models (adjusted for age, gender, and APOE4 genotype) were used to explore the association between binary change in cognitive scores for each neuropsychological testing (dependent variable) and AD-RAI, BrainAGE, and NPH score respectively. To explore the added value of these AI MRI markers compared to a set of already established AD imaging markers in brain aging, we also built logistic regression models including each AI marker and amyloid and FDG PET positivity, MTA, CMB, Fazekas score at baseline. The added value of AI markers as compared to conventional AD imaging markers was assessed using the likelihood-ratio (LR) test and receiver operating characteristic (ROC) curves, along with the Delong tests of equality of ROC areas as implemented in Stata’s “roccomp” command. The significance level was set at *p* < 0.05. All analyses were performed with Stata release 17.0.

## Results

### Clinical and imaging MRI descriptive data

There were no significant gender-related differences in clinical and imaging parameters at baseline. At follow-up, 38% of cases remained cognitively stable or improved their performances. Of importance, none of our cases evolved to MCI during the follow-up period. As expected in this cohort of healthy controls, only 26.5% of cases showed abnormal FDG-PET patterns. Amyloid positivity was present in 23.8% of cases. In the same line, the median NPH probability was very low at the 3.6% interquartile range (1.3–11.0%) (Table 1).

**Table 1:** Gender-related distribution of clinical and imaging variables at baseline

	Female	Male	Total	*p* value
N	46	34	80	
Age at baseline	73.6 ± 3.8	73.1 ± 3.2	73.4 ± 3.5	0.655
MMSE at baseline	28.6 ± 1.1	28.8 ± 1.0	28.7 ± 1.1	0.205
ApoE4	9 (19.6%)	6 (17.7%)	15 (18.8%)	0.828
Digit Span (lower = worse)	36 (75.0%)	24 (75.0%)	60 (75.0%)	1.000
Shapes test (lower = worse)	31 (85.4%)	41 (96.9%)	72 (90.0%)	0.135
Corsi score (lower = worse)	14 (30.4%)	10 (29.4%)	24 (30.0%)	0.668
Trail A time (higher = worse)	12 (25.0%)	10 (31.3%)	22 (27.5%)	0.613
Trail B time (higher = worse)	13 (28.3%)	6 (17.6%)	19 (23.8%)	0.821
FDG PET abnormal	13 (28.3%)	8 (23.5%)	21 (26.3%)	1.000
Amyloid positivity	12 (26.1%)	7 (20.6%)	19 (23.8%)	0.607
Right mesial temporal lobe atrophy	35 (76.1%)	24 (70.6%)	59 (73.8%)	0.806
Fazekas score				0.787
0	18 (39.1%)	16 (47.1%)	34 (42.5%)	
1	20 (43.5%)	12 (35.3%)	32 (40.0%)	
2/3	8 (17.4%)	6 (17.6%)	14 (17.5%)	
Number of CMBs				0.176
0	23 (50.0%)	24 (70.6%)	47 (58.8%)	
1	10 (21.7%)	6 (17.6%)	16 (20.0%)	
2–3	11 (23.9%)	3 (8.8%)	14 (17.5%)	
4–6	2 (4.3%)	1 (2.9%)	3 (3.8%)	

### Imaging-based prediction of neuropsychological parameters

#### Corsi score

**Fig. 1:**
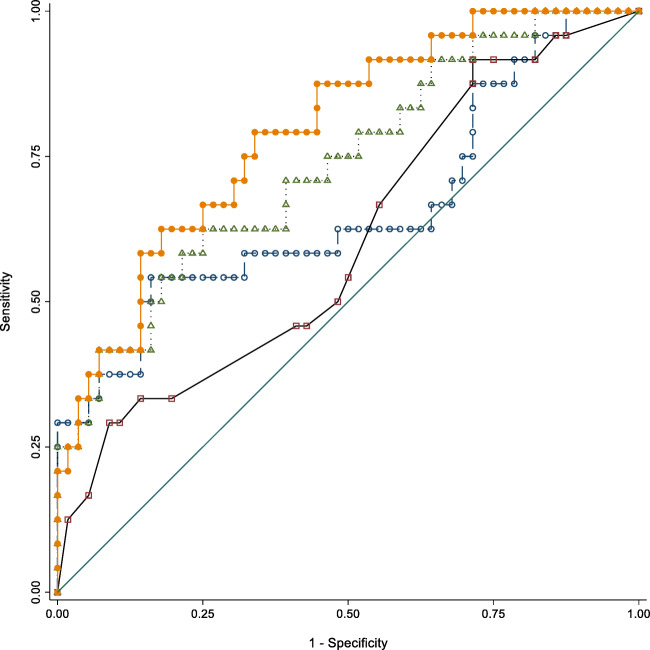
Area under the ROC curves for Corsi score. Model 1 (open square): AD-RAI [0.660 (95% CI: 0.514–0.806)]; Model 2 (open circle): conventional MRI variables [0.604 (95% CI: 0.467–0.741)]; Model 3 (open triangle): All MRI variables without amyloid positivity [0.735 (95% CI: 0.613–0.858)]; Model 4 (filled circle): All MRI variables and amyloid positivity [0.793 (95% CI: 0.688–0.898)]. See text for details

Among the neuropsychological tests used, the Corsi score (visuospatial memory) decline was associated with the AD-RAI score at baseline (OR: 1.07 [CI: 1.02, 1.12], *p* = 0.002) (Fig. 1). This single AI marker explained 14.7% of the cognitive variability in univariate regression models. After adjustment for age, gender, and APOE4 genotype, the percentage of cognitive variability explained by the model reaches 21.8%, but none of the adjusting variables are significant (supplement Table 1). This AD-RAI score was not related to the Corsi score change over time. Importantly, when amyloid positivity, FDG-PET positivity, MTA, CMB, and Fazekas score were included in the regression models, the AD-RAI score remained the sole valid predictor of the cognitive outcome. The addition of the AD-RAI score led to a significant increase in the percentage of cognitive variability explained by the regression model (from 3.4 to 16.7%, likelihood ratio, *p* = 0.0003) (Table 2). Of importance, amyloid positivity was marginally significant (*p* = 0.060) but its addition improved significantly the percentage of cognitive variability explained by the model reaching 21.4% (likelihood ratio, *p* = 0.0335).

**Table 2:** Multiple logistic regression analysis with Corsi score as the dependent variable and imaging parameters as independent predictors. Note that only AD-RAI is significantly related to the cognitive outcome

	OR adjusted	95% CI	*p* value
Amyloid positivity	0.20	[0.04, 1.07]	0.060
AD-RAI [%]	1.08	[1.03, 1.13]	0.002
Fazekas	1.40	[0.68, 2.89]	0.358
MTA	2.44	[0.59, 10.13]	0.220
Number of CMBs	0.98	[0.58, 1.66]	0.943

#### Trail Making B score

**Fig. 2:**
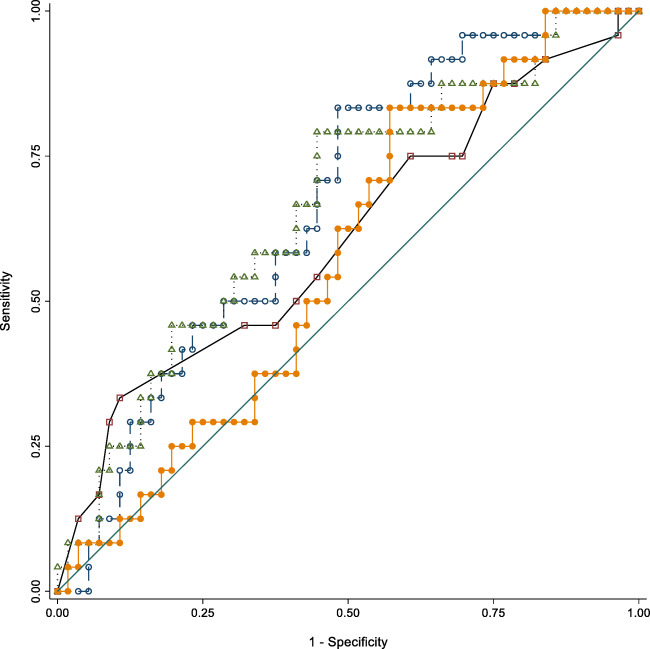
Areas under the ROC curves for Trail Making B score. Model 1 (open square): BrainAGE alone; Model 2 (open circle): MRI variables without BrainAGE and Amyloid positivity; Model 3 (open triangle): MRI variables without amyloid positivity; Model 4 (filled circle): All variables. Model 3 has an AUC which is significantly different from 0.5

BrainAGE score at baseline was associated with Trail Making B (executive functions) score worsening upon follow-up (OR: 1.28 [CI: 1.06, 1.56], *p* = 0.013) (Fig. 2). This single AI marker explained 8.3% of the cognitive variability in univariate regression models. After adjustment for age, gender, and APOE4 genotype, the percentage of cognitive variability explained by the model reaches 13.8%, but none of the adjusting variables is significant (supplement Table 2).

As for the Corsi score, the APOE4 genotype was not related to the cognitive outcome. Among the conventional MRI markers, only the Fazekas score at baseline was positively related to the cognitive outcome (OR: 2.38 [CI: 1.14, 4.97, *p* < 0.05]) explaining 8.7% of the cognitive variability. The addition of the BrainAGE score led to a significant increase in the percentage of cognitive variability explained by the regression model (from 8.7 to 14.0%, likelihood ratio *p* = 0.0311; Table 3). When amyloid and FDG positivity were added to the regression model, only the first was strongly associated with Trail Making B worsening. The addition of amyloid positivity improved significantly the percentage of cognitive variability explained by the model reaching 21.8% (likelihood ratio, *p* = 0.0089). In this global model, the Fazekas score becomes marginally significant (*p* = 0.058).

**Table 3:** Multiple logistic regression analysis with Trail Making B score as a dependent variable and imaging parameters as independent predictors. Note that amyloid positivity, BrainAge, and Fazekas scores were significantly related to the cognitive outcome

	OR adjusted	95% CI	*p* value
Amyloid positivity	5.23	[1.47, 18.59]	0.011
BrainAGE	1.30	[1.03, 1.63]	0.027
Fazekas	2.24	[0.97, 5.15]	0.058
MTA	1.60	[0.34, 7.46]	0.548
Number of CMBs	0.80	[0.41, 1.54]	0.498

There were no other statistically significant associations between these AI markers at baseline and cognitive parameters in the present study. In particular, neither AD-index nor BrainAGE scores were related to MMSE score decrement (global cognition). In addition, the NPH score at baseline was not associated with cognitive changes upon follow-up in this cohort of elderly controls.

### ROC curve analyses

For Corsi score, the areas under the ROC were of 0.604 (95% CI: 0.467–0.741) (conventional MRI variables), 0.660 (95% CI: 0.514–0.806) (AD-RAI score), and 0.735 (95% CI: 0.613–0.858) (conventional MRI variables and AD-RAI score). When adding amyloid positivity in the last model, the area under the ROC was 0.793 (95% CI: 0.688–0.898). There was a statistically significant difference between the areas under the ROC curves (*p* < 0.0001). The combination of all MRI markers plus amyloid positivity performed significantly higher compared to conventional MRI variables according to both the LR test (LR *p* = 0.0002) and the ROC curve comparison (*p* = 0.0053). The same combination performed significantly higher compared to the AD-RAI score alone according to the ROC curve comparison (*p* = 0.0279)., but not with the LR test (LR *p* = 0.1625).

For Trail Making B score, the areas under the ROC were of 0.609 (95% CI: 0.468–0.749) (for conventional MRI variables), 0.666 (BrainAge alone) (95% CI: 0.544–0.788), and 0.664 (95% CI: 0.534–0.793) (conventional MRI variables and BrainAge). When adding amyloid positivity in the last model, the area under the ROC was 0.574 (95% CI: 0.444–0.704). There was a statistically significant difference among the areas under the ROC curves (*p* = 0.0317).

BrainAge alone and the combination of conventional MRI variables and BrainAge performed better than chance with an area under the ROC that differed significantly from the non-discriminant area of 0.5.

Adding the combination of conventional MRI variables to BrainAge alone did not improve the discriminating power of the latter according to both the LR test (LR *p* = 0.1675) and the ROC curve comparison (*p* = 0.9652).

## Discussion

The present data reveal that two among the newly proposed AI MRI markers have a limited but still significant added value in predicting the cognitive trajectory in elderly controls. Although they are not related to global measures of cognition such as the MMSE score, AD-RAI index, and BrainAGE score are associated with the decrement of visuospatial and executive function measures in cognitively preserved elders. Of importance, both markers have a significant added value when compared to widely used MRI markers of brain aging such as the Fazekas score, MTA, and number of CMB. For the NPH-score, no significant association with neuropsychological performances in old age was found.

Three recent studies explored the relevance of AD-RAI as a diagnostic marker for AD [23], for predicting the MCI conversion to AD [44], and for the detection of preclinical and prodromal AD [45]. In a series of 50 AD patients and 50 controls, the AD-RAI use led to a diagnostic accuracy of 0.91 outperforming the volume of any single brain structure measured [23]. An AD-RAI cut-off of 0.5 had a better performance to differentiate MCI converters from non-converters [44]. Moreover, this parameter achieved the best metrics (compared to conventional MRI markers) in detecting elderly controls positive for amyloid and tau (A+T+) [45]. Our results go beyond these observations by showing that AD-RAI is an independent predictor of visuospatial memory decline in the present cohort. MTA, Fazekas score, and CMB were all unable to predict such subtle changes in cognitive performances in these elderly controls. Both likelihood-ratio test and receiver operating characteristic (ROC) curves demonstrated that the addition of AD-RAI significantly increases the ability to predict the decline of visuospatial memory. Of importance, even in these cases with low amyloid burden, the combination of amyloid positivity and MRI markers including AD-RAI provides the best performance in predicting the cognitive outcome.

In the last decade, BrainAGE was established as an image-based biomarker to capture the general aging processes of the brain [40]. It was shown to be altered in various neuropsychiatric conditions, most prominently in age-associated disorders such as Alzheimer’s disease (AD) [27]. Moreover, BrainAGE was able to predict the risk of incident dementia in a cohort with mild cognitive impairment [46] and in the general population [47]. In this latter study, the incidence of dementia was related to the prediction of brain age based on MRI-derived gray matter in 3688 dementia-free cases from the Rotterdam study. This association persisted in logistic regression models adjusted for white matter hyperintensities and hippocampal volume. Our data are less impressive since the BrainAGE score was not related to the evolution of global cognition upon 4.5-year follow-up. However, and unlike conventional MRI markers, BrainAGE was an independent predictor of declining performance in executive functions (Trail Making Test B). This imaging parameter was an independent predictor of executive function performance decrease after adjusting for Fazekas score, CMB, and MTA at baseline. In particular, likelihood ratio test revealed that the addition of Brain Age scores significantly improves the percentage of cognitive variability explained by the regression models. This was also the case for amyloid positivity. However, only the model that included all conventional MRI markers and Brain Age was statistically significant in terms of area under the ROC indicating that the contribution of BrainAge as a single imaging marker in the prediction of executive function decline in our cohort remains modest.

Several reasons may explain the discrepancy between the present data and the recent observations by Wang et al [47]. First, none of our cases evolved to MCI during the follow-up period pointing to the presence of cognitive resilience in our sample without significant vascular burden. The cognitive outcome was defined as the binary change (decrement versus improvement or stability) in neuropsychological scores without reference to incident dementia. Furthermore, since the BrainAGE score used in our study—like most other approaches—is based on T1-weighted imaging data only, it can be assumed that age-related changes are predominantly found in other sequences (such as white matter lesions as assessed on FLAIR imaging) might be neglected. In addition, BrainAGE is also influenced substantially by parameters such as lifestyle factors (e.g. smoking [48]), genotypic variations (e.g. APOE status) [46], or early neurodevelopmental influences such as preterm birth [49] that cannot be taken into account given the limited sample of this study. Despite these differences, the present findings imply that the BrainAGE score remains a useful predictor of longitudinal changes in executive functions during the very early stages of the aging process.

Recent studies suggest that imaging markers of NPH, which imply cerebrospinal fluid disorders, might be an independent imaging markers of non-AD pathophysiology [29]. The NPH index is an operator-independent imaging marker of possible cerebrospinal fluid disorder that has been proposed as a possible candidate for the prediction of cognitively asymptomatic cases at risk for AD [28]. In our cohort, we could not demonstrate significant associations between NPH index at baseline and both global cognition and neuropsychological test changes—taking into account as a limitation that NPH index values were very low in our cohort. The NPH index might provide more valuable results in other cohorts with a higher proportion of cases with elevated NPH index.

Among the strengths of the present study, one should note the detailed cognitive analysis and use of multivariable models that allow for defining the added value of the newly proposed AI imaging parameters compared to traditional imaging markers of brain aging. Several limitations should be considered. The small sample size limits the number of imaging predictors that can be simultaneously included in regression models, we excluded all of the cases with significant vascular burden in order to focus on the AD trajectory in brain aging. Thus, our cases are not representative of the whole spectrum of brain aging. Despite their recruitment in the community, they display no or very mild vascular pathology and a relatively high level of education. The assessment of white matter lesions was made using T2w sequences and not the traditional FLAIR images. However, it is quite rare that e.g. Fazekas 1 in FLAIR is considered Fazekas 2 in T2w (or the inverse) by a well-trained radiologist. Most importantly, the low occurrence of amyloid positivity and brain hypometabolism in this sample indicated that the number of incipient AD cases may be lower than expected in a community-based sample of elderly persons. It is thus likely that the added value of AI measures may increase when cases with a higher amyloid burden are considered. Based on our observations, future studies in mixed samples are clearly warranted to define the relevance of the AI-MRI measures in the field of brain aging and AD.

The AI-based AD-RAI index and BrainAGE scores have limited but significant added value in predicting the subsequent cognitive decline in elderly controls when compared to the established visual MRI markers of brain aging, notably MTA, Fazekas score, and number of CMBs.

## Supplementary information

ESM 1 (DOCX 18 kb)

## Abbreviations

AD: Alzheimer disease
ADRAI: Alzheimer Resemblance Atrophy Index
AI: Artificial intelligence
BrainAGE: Brain Age Gap Estimate
CSF: Cerebrospinal fluid
FLAIR: Fluid attenuated inversion recovery
MR: Magnetic resonance
MTA: Mesio-temporal atrophy
NPH: Normal pressure hydrocephalus
WML: White matter lesions

## Declarations

### Guarantor

The scientific guarantor of this publication is Sven HALLER.

### Statistics and biometry

One of the authors (FR HERRMANN) has significant statistical expertise.

### Study subjects or cohorts overlap

Some study subjects or cohorts have been previously reported in various papers of the group (see citations).

### Methodology

• prospective

• cross-sectional study

• performed at one institution
