# Supplementary material for: Alzheimer resemblance atrophy index, BrainAGE, and normal pressure hydrocephalus score in the prediction of subtle cognitive decline: added value compared to existing MR imaging markers
Source: Eur Radiol. 2022 Apr 29;32(11):7833–42. doi: 10.1007/s00330-022-08798-0 (PMC9668758; doi:10.1007/s00330-022-08798-0)
Supplement: Supplementary file 1 — (DOCX 18 kb) [file 330_2022_8798_MOESM1_ESM.docx]

**Supplement Table 1: Simple and multiple logistic regression analysis with Corsi score as the dependent variable and AD-RAI as independent predictor. Without and with adjustment for age, sex and ApoE4.**

|  |  |  |  |  |  |  |  |
| --- | --- | --- | --- | --- | --- | --- | --- |
|  | **OR** | **95% CI** | **p-value** |  | **OR adjusted** | **95% CI** | **p-value** |
|  |  |  |  |  |  |  |  |
| AD-RAI [%] | 1.07 | [1.02, 1.12] | 0.002 |  | 1.08 | [1.02, 1.15] | 0.007 |
| Male sex |  |  |  |  | 2.15 | [0.63, 7.40] | 0.225 |
| Age |  |  |  |  | 1.04 | [0.88, 1.24] | 0.610 |
| ApoE4 |  |  |  |  | 0.12 | [0.01, 1.20] | 0.071 |
|  |  |  |  |  |  |  |  |

**Supplement Table 2: Simple and multiple logistic regression analysis with Trail Making B score as the dependent variable and Brain Age as independent predictor. Without and with adjustment for age, sex and ApoE4.**

|  |  |  |  |  |  |  |  |
| --- | --- | --- | --- | --- | --- | --- | --- |
|  | **OR** | **95% CI** | **p-value** |  | **OR adjusted** | **95% CI** | **p-value** |
|  |  |  |  |  |  |  |  |
| BrainAGE | 1.28 | [1.06, 1.56] | 0.013 |  | 1.32 | [1.07, 1.61] | 0.008 |
| Male sex |  |  |  |  | 0.52 | [0.16, 1.68] | 0.272 |
| Age |  |  |  |  | 1.15 | [0.99, 1.33] | 0.073 |
| ApoE4 |  |  |  |  | 0.86 | [0.18, 4.08] | 0.851 |
|  |  |  |  |  |  |  |  |
